# Supplementary material for: Are carbon emissions trading and green financial instruments synergistic? -Comprehensive quantitative research based on content analysis
Source: PLoS One. 2024 Mar 7;19(3):e0298601. doi: 10.1371/journal.pone.0298601 (PMC10919601; doi:10.1371/journal.pone.0298601)
Supplement: S1 Data — (ZIP) [file pone.0298601.s001.zip › Data/地级市Z_F工作报告.zip!/╕╩╦α/╒┼╥┤╩╨/└┤╫╘╣1⁄2╓┌║┼í╛┬φ┐╦╩2╛▌═°í┐_2019.docx]

各位代表：

现在，我代表市人民政府向大会报告工作，请予审议，并请各位政协委员和列席人员提出意见。

2018年工作回顾

刚刚过去的一年，在省委、省政府和市委的坚强领导下，我们坚持以习近平新时代中国特色社会主义思想为指导，认真贯彻党的十九大及十九届二中、三中全会精神，全面落实习近平总书记视察甘肃重要讲话和“八个着力”重要指示精神及中央、省委省政府和市委一系列决策部署，坚持稳中求进工作总基调，按照高质量发展要求，统筹推进稳增长、促改革、调结构、惠民生、防风险、保生态各项工作，全市呈现出经济平稳运行、社会和谐稳定的良好局面。

—— 综合经济实力持续增强。面对经济下行压力较大的形势，以强烈的发展愿望和责任担当，采取既利当前又惠长远的举措，积极应对，科学调度，力促经济回稳向好，综合实力有了新的提升。全年实现生产总值407.71亿元，增长5.8%；开工建设项目642项，完成固定资产投资218.8亿元，增长12.12%，增幅居全省第2位；完成大口径财政收入60.64亿元、一般公共预算收入27.84亿元，分别增长2.61%和5.76%；实现社会消费品零售总额176.05亿元，增长6.9%；城镇、农村居民人均可支配收入达25267元和13710元，分别增长8.4%和8.7%；存款余额670.64亿元，增长9.15%，存贷比达94.24%。三次产业结构调整为21.86:22.27:55.87，现代农业转型加快，工业经济止滑回升，以旅游文化为龙头的第三产业贡献率稳步提高，经济结构更趋优化，高质量发展综合排名全省第5位。

—— 优势主导产业提质增效。坚持旅游文化龙头产业地位不动摇，以延链补链项目建设和管理服务水平提升为重中之重，深入推进“四大行动”，大力发展旅游新业态、新模式。新创建4A级景区3家，丹霞景区5A级创建进入终评阶段。新建续建七彩古街、沙漠胡杨林、彩虹山丹、金山公园等重点旅游项目85项，丹霞口民俗文化旅游村、张掖国际露营基地等建成运营，完成投资40亿元。新建改建旅游厕所93座。成功举办祁连山国际超百公里挑战赛、中国汽车拉力赛等大型赛事活动26项，张掖连续两年获评“中国体育旅游十佳目的地”，户外运动体验区名声愈加响亮。建成智慧旅游管理、营销、服务三大平台，全面开展旅游市场秩序专项整治“利剑行动”，查处违规旅游企业15家，管理服务水平进一步提升。持续开展全民旅游宣传活动，60多家主流媒体深度宣传报道张掖，举办各类旅游推介活动20场次，开通旅游专列278趟、正线航班和直航包机799班次。旅游人次达到3178万、旅游综合收入达到210.7亿元，分别增长22.3%和33.9%，提前两年实现预期目标，旅游对经济社会发展的贡献率和对外影响力不断提升。

坚持建设绿色有机农产品基地目标不动摇，紧扣规模、链条、品牌、效益等关键环节，大力推动优势产业延链增效、特色产业扩量提质，“三元双向”循环模式更趋成熟，质量兴农、品牌强农、绿色富农迈出新步伐。大力发展戈壁农业，海升现代智能温室工业化栽培生态示范及甘州明永滩、高台新绿达、临泽扎尔墩等10个千亩示范园开工建设，辐射带动建成非耕地设施农业2.8万亩，完成投资11亿元。深入实施“四个百万”工程，新增蔬菜5.1万亩、中药材6.6万亩，食用菌产量达到7.4万吨，新改扩建标准化规模养殖场区115个，肉牛、奶牛饲养量分别达到93.8万头和4.3万头，人工种草面积达到136万亩，优势特色产业规模持续扩大。着力延伸产业链，临泽鸿泽食用菌、山丹祥永麦芽等16个重点加工项目建成投产，金沙5万吨脱水蔬菜、三禾食用菌、丰大玉米种子加工等重点项目加快建设，完成投资19.73亿元，新增农产品加工能力5.3万吨。持续推进农业标准化建设，新认证“三品一标”农产品79个，高台被确定为全省唯一、全国首批农业绿色发展试点先行区，临泽获评国家级农产品质量安全县，民乐农村产业融合发展示范园被认定为首批国家级示范园，28个产品获评甘肃名牌产品，“金张掖”农产品品牌更加响亮。

坚持资源循环利用、企业集约发展的生态工业发展方向不动摇，加快工业项目建设，支持企业科技创新，强化要素保障服务，力促工业经济回暖向好。张掖经济技术开发区循环经济示范园污水处理厂、高台盐池工业园区热源厂等一批基础设施项目加快建设，临泽通航产业园、凹凸棒产业园初具规模，民乐生态工业园区获批国家第三批绿色制造园区，园区功能进一步完善。甘州区智能终端产业科技城加快建设，融信、龙起半导体等智能电子企业入园发展，实现当年引进、当年投产。开工建设投资千万元以上重点工业项目100项，鼎丰源凹凸棒新材料加工、海牙沟矿泉水开发等71项建成，晋昌源20万吨中温焦油加氢、天域绿谷三产融合等29项加快建设，完成投资34.1亿元。启动“十强双百”企业振兴计划，实施停产企业“一企一策”方案，落实电价补贴、应急周转金支持等帮扶措施，52户企业成功复产，工业经济呈现恢复性增长。实现规上工业增加值34.44亿元，增长4.3%，较上年提高21.4个百分点。

—— 城乡基础设施显著改善。坚持完善公共服务设施与美化环境齐抓、丰富内涵与塑造特色并重，持续加强基础设施建设，城乡面貌不断改善，发展后劲进一步增强。启动新一轮《张掖市城市总体规划》及《张掖市历史文化名城保护规划》修编工作，完成城东片区和东北郊生态科技产业园区控制性详规等编制任务。市区国道312线、省道213线等五个出城口提升改造工程加快推进，临泽、高台、山丹、肃南县城出城口提升改造工程全面完工，改拓建市区及各县城区道路50条41公里，市区东南西北四条大街、西南二环改造工程完成投用，市区21条街道沿街既有建筑风貌色彩改造工程全面完成，新增城市绿地108公顷，城市整体形象进一步提升。临泽县城新水源地及供水厂、山丹县城污水处理厂提标技改、市区集中供热备用热源提标改造等一批市政基础设施项目建成投运，全市生活污水处理厂运行负荷率达89.6%，新增集中供热面积274万平方米。市生活垃圾焚烧发电厂二期工程加快建设，全市城市生活垃圾收集转运处理一体化机制初步建立。

坚持统筹规划、分步实施、重点示范，启动实施乡村振兴“两带四区四线”示范建设。张肃公路沿线甘州双塔、速展等重点村拆迁改造工作取得突破性进展，临泽梨园新村三期、丹霞乡情家园、14万亩生态公益林补水等重点工程有序推进，肃南白银乡蒙古族特色村寨、康乐镇裕固族民宿度假区加快建设；张民公路拓建工程基本完工，土地整理、旧房拆除加快推进。73个“美丽乡村”示范村和102个“万村整洁”示范村建设成效明显，一批农村文化、科技等公建项目及道路改造、给排水、垃圾处理工程建成投用，完成投资8.34亿元。拆除破旧空置房屋3639户，持续开展改炕改厕改厨等专项行动，农村人居环境进一步改善。启动实施“双十双百”乡村旅游示范工程，评定市级专业旅游村12个，山丹硖口村列入国家第五批传统村落名录，甘州前进、临泽南台等17个村被列为全省乡村旅游示范村，屋兰古镇黄河灯阵、喀尔喀小镇等一批重点乡村旅游项目建成投用，多点支撑、全域拓展的乡村旅游格局加快形成。

加快推进交通水利电力通讯等基础设施建设，有效破解融资投资、征地拆迁、建设运营等制约难题，一批大项目建成投用，基础设施条件持续改善。六东一级、平甘一级、G312临泽过境段、S590山丹至马场二场及月牙湖、大湖湾旅游公路等9项241公里重点公路建成通车，张掖西站综合客运枢纽、丹霞景区客运站全面完工，新改建农村公路及安防工程729公里，张扁高速、G213肃南至祁连公路、G30丹霞互通立交、G312马营口至山丹西公路等重点工程加快推进，完成投资45.18亿元。黑河城区生态治理一期、白石崖水库基本建成，山城河、金山水库进展顺利，高效节水灌溉示范、农村饮水安全巩固提升、中小河流治理、灌区节水改造等重点工程有序推进，完成投资9亿元。整理土地15万亩，新增耕地1.27万亩，完成投资1.2亿元。新建通信光缆4129公里、4G基站928个，37个4G基站未覆盖行政村纳入国家电信普遍服务试点建设支持范围，城市运行管理及应急指挥中心建成投用，智慧城市完成大数据中心建设和框架搭建任务；张掖750千伏输变电、甘州330千伏变电站、南华330千伏牵引变改造工程开工建设，通讯电力保障能力进一步增强。

—— 民生保障水平稳步提高。坚持多办利民实事、多解民生难事，坚决兜住民生底线，群众获得感、幸福感不断提升。制定完善“一户一策”精准脱贫计划和巩固提高计划，筹措落实财政扶贫专项资金2.6亿元。引导成立农民专业合作社440家，带动贫困村、贫困户发展特色产业7.8万亩。65个贫困村全部开通沥青（水泥）通村通社道路，13821户51375人饮水安全问题得到解决。精心实施易地扶贫搬迁项目，2017年度2550户8706人安置房交付使用，2018年度157户619人安置房完成主体工程。就业、教育、健康、电商、生态等领域扶贫成效显著，1201户3986人实现脱贫，剩余38个贫困村全部验收退出，5个“插花型”贫困县区全部脱贫摘帽，全市贫困发生率下降至1.15%。

突出基础配套，强化规范管理，社会各项事业全面协调发展。张掖中学顺利搬入新校区，甘州思源实验学校建成投用，青西小学、育才中学恢复办学。投资2.7亿元，实施学前教育、“改薄”项目、教育现代化推进工程等五大类157个项目，办学条件进一步改善。学前三年毛入园率达99.34%、普惠性幼儿园覆盖率达91%，分别高于国家2020年目标14.34和11个百分点。普通高考本科上线率达83.06%，高于全省14.94个百分点；总录取率达96%，高于全省14.35个百分点，连续七年居全省第一。加快建设“健康张掖”，公立医院薪酬制度改革试点、药品零差率销售、分级诊疗、药品购销“两票制”、家庭医生签约服务等医药卫生体制改革深入推进，“先诊疗、后付费”及基本医保、大病保险、民政救助“一站式”结报制度和“一人一策”健康帮扶措施有效落实，城乡居民医疗费用补偿比例提高到73.02%；市第二人民医院一期、山丹新区中医院及高台县妇幼保健院建成投用，甘州、临泽、民乐人民医院业务用房加快建设；持续加强公共卫生服务体系建设，疾病防控工作规范有序，卫生保健水平不断提升。全面两孩政策稳步实施，落实人口优惠政策补助资金8700万元，惠及27.8万人次。深入推进国家现代公共文化服务体系示范区巩固提升和扩大文化消费试点城市建设，全省首家数字文化馆建成投用，县区重点文化场馆项目加快实施，累计建成博物馆101座，高台明长城保护修缮等13项重点文物保护工程有序推进，七一剧院、市文化馆投入使用，市丝绸之路文化艺术中心基本建成；“2018张掖·敦煌写经书法”艺术节纳入第三届敦煌文博会并成功举办，裕固族民歌保护传承项目通过国家评审，《民乐情》荣获全国现实题材优秀剧目并在全省巡演，5部文艺作品获“敦煌文艺奖”。建成大型公共体育场、足球场、游泳馆、健身中心7处及健身步道150公里，群众健身环境日益改善。35家企业被新认定为高新技术企业，国家农业科技园区、甘州区及民乐县省级农业科技园区通过验收。

持续增强民生保障能力，支出民生资金133.4亿元，增长5.74%，占财政总支出的82.75%。强化全方位就业服务，城镇新增就业2.52万人，城镇登记失业率控制在2.6%。加强技能培训，输转劳动力29.3万人。推进社保扩面征缴，城乡居民基本养老保险续保率达95%。完善城乡居民基本医疗保险制度，全面实现异地就医直接结算，住院合规费用报销比例达75%以上。筹资756万元，为7.47万名农村妇女进行“两癌”免费检查。落实中央及省级补助资金6.77亿元，争取政策性棚改贷款和专项债券40.93亿元，开工建设棚户区16129套（户），基本建成12962套（户），发放住房租赁补贴4918户。改造安装老旧住宅小区电梯20部。城乡低保和特困供养保障标准均提高7.6%，发放低保等困难群众基本生活和医疗救助资金4.62亿元。继续推进职工互助保障计划，统筹做好城镇困难职工解困脱困工作。新建续建残疾人康复、托养机构6家。积极开展养老院服务质量建设专项行动，为132个日间照料中心补助经费259万元，市级居家养老服务信息平台、临泽居家养老服务中心建成投用，甘州孔文、民乐尚福佳苑等社会养老机构加快建设，养老服务体系渐趋完善。

—— 改革创新活力加快释放。坚持问题导向，着力推动重点领域和关键环节改革落地见效，市场主体活力、经济发展动力持续增强。全面推行“四办”改革，取消调整行政审批事项26项，公布取消证明事项72项，市县两级公布“最多跑一次”办事事项2300项，建成60个乡镇、42个社区便民服务中心和776个行政村便民服务点，市县行政许可事项信息全部与甘肃政务服务网加载对接，以“一个体系”“两条通道”“三级延伸”为核心的政务服务体系基本建成。市县两级共享公共资源交易网及电子服务、交易、监管三个平台上线运行。多证合一、企业简易注销、政银便民通、证照分离等便利化商事制度改革措施全面落实，企业和群众满意度明显提升，新增各类市场主体1.49万户。环评审批制度改革做法被国务院办公厅《政务公开工作交流》转发，向全国推广，环评改革的效能和示范效应持续扩大。全面深化职称制度改革，专业技术人才扎根基层、创新创业积极性明显增强。

着力巩固提升“双创示范”成果，累计建成各类创业创新基地、空间178万平方米，入驻入孵企业4926户，新增就业5.9万人，“双创示范”工作获国务院和省政府通报表扬，张掖入选《半月谈》2018十佳最具绿色投资价值城市和《环球时报》2018中国最具投资吸引力城市，高台获评全省唯一全国创新型县。积极推进农村“三变”改革，154个试点村辐射带动农户2.9万户，实现入股分红1989万元，带动村集体增收619万元。农村集体清产核资工作全面完成，清理资金4.52亿元、资产56亿元及耕地、林地、“四荒地”2775万亩。农村产权交易市场持续活跃，完成土地流转交易733宗、2.5亿元；办理农村产权交易鉴证378份，265户经营主体获得贷款16.2亿元。设立甘肃金控张掖融资担保公司，政策性担保体系进一步完善。市属五大国有投融资平台融资10.08亿元，完成投资35.24亿元。创新保险扶贫模式，平安产险“免息免担保”产业扶贫项目落地实施。五色建材等国企改革有序推进，市级机关事业单位农场管理体制改革任务全面完成，国有林场改革通过省级评估验收。全国地方公车改革现场会在张掖召开，推广介绍了张掖的做法和经验。

持续推进对外开放，组织开展各类赴外招商活动40多场次，邀请60多批次500多人来张考察，成功举办第五届“张交会”、第二届陇药博览会等大型招商节会，新签约招商项目183项，总投资262亿元。新建续建招商项目317项，到位资金223.8亿元，增幅居全省前列。实现自营出口1.87亿元，出口供货22亿元。新增农产品出口备案基地5.5万亩，累计达到25.8万亩。供港澳蔬菜基地达到1.3万亩，首发中新南向通道张掖农产品国际专列，农业“走出去”迈出新步伐。持续完善商流市场体系，开工建设25个商流重点项目，完成投资10.7亿元。全省首家种子交易中心获批，甘肃东方种业交易中心股份公司启动运营。全面开展物流市场秩序整治专项行动，制定出台城区物流体系发展规划。举办金张掖农特产品网上展销会，京东商城张掖馆上线运营。新发展电商企业70多家、网店微店700多个，从业人数达1.4万人，电商交易额达35亿元。

—— 生态文明建设实现突破。统筹推进生态环境保护和重点领域治理，全方位、全地域、全过程整治保护修复，生态保护红线、环境质量底线、资源利用上线守得更紧更牢。坚持分类施策、压茬推进，179项祁连山生态环境问题全部完成现场整治任务，77项矿业权全部退出，7座退出水电站全部完成设施设备拆除清理和生态环境恢复任务，3项旅游项目全部拆除设施。草原减畜任务全面完成，1571万亩草原实施禁牧，342.59万亩林草“一地两证”问题得到稳妥解决。搬迁农牧民生活生产有效保障，搬迁区域生态基本恢复。祁连山国家公园体制试点建设稳步推进，管理机构移交等工作基本完成。持续加强黑河湿地保护，保护区范围内334项生态环境问题全部整改销号。持续开展祁连山、黑河湿地自然保护区外围区域生态环境问题排查治理，现场整治215个矿山项目，36座引水式电站生态基流下泄监控设施及信息化平台建成投用。

加快实施山水林田湖草生态保护修复工程和祁连山保护修复综合治理规划项目，累计完成投资38.2亿元，是建国以来祁连山生态保护投资额最大的一年。祁连山国家公园和黑河生态带、交通大林带、城市绿化带“一园三带”造林绿化示范建设完成营造林31.1万亩，带动大规模国土绿化50.8万亩，超过前三年总和。

深入开展大气、水、土壤污染防治攻坚。整治燃煤锅炉549台5836蒸吨，建成5处煤炭集中交易市场、74个二级配送网点，183个施工场地全部落实防尘抑尘措施，改造土炕4万多铺，扎实开展烧荒整治行动，空气质量名列全省第二。市县乡村四级“河湖长”体系全面建立，严厉打击非法排污、采砂、围垦、乱倒垃圾等破坏水生态行为，市区4条黑臭水体治理工程全面完成，水源地保护进一步加强，水生态环境质量持续提升。深入实施农药、化肥、地膜减量化等专项行动，加强土壤环境监察执法，土壤资源安全保障能力不断提高。全面推进全域无垃圾行动，“户分类、村收集、乡镇转运、县区处理”的城乡生活垃圾处置体系基本建立，市垃圾焚烧发电厂处置生活垃圾8.5万吨。在全省率先建成投用“一库八网三平台”生态环境监测网络，“天上看、地上查、网上管”的立体化生态环境监管格局基本形成，生态环境、重点污染源及环境质量实现全域监测，获评全国2018年智慧环保十大创新案例。

—— 政府服务效能明显提升。深入落实“三重”“三一”工作措施，健全“市级政府决策担责、县区部门落实执行”的工作机制，政府决策科学化、执行力明显提升。建立完善市政府议事会议制度，先后就金融风险防控、非公经济发展、物流体系建设进行专题深度研究，政府决策针对性、时效性进一步提高。强化企业安全生产主体责任，加大风险分级管控和隐患排查治理，事故起数、伤亡人数大幅下降，安全生产形势持续稳定。深入推进法治张掖建设，依法行政工作取得明显成效。大力整治非法金融活动，立案查处非法吸收公众存款案12起。深入开展扫黑除恶专项斗争，破获涉黑涉恶案件85起，打掉恶势力犯罪团伙12个。积极创建全国禁毒示范城市，毒品综合治理能力不断提升。持续推进阳光信访、责任信访、法治信访建设，矛盾纠纷化解扎实有效。积极推进民族团结进步创建，依法加强宗教事务管理，民族团结、宗教和顺局面不断巩固。健全完善防灾减灾救灾体制机制，应急处置能力不断提升。严格落实“四个最严”“四有两责”要求，食品药品安全监管进一步加强。扎实开展双拥共建工作，优抚安置政策全面落实。深入开展“转变作风改善发展环境建设年”活动，政府系统工作作风持续好转。认真贯彻全面从严治党要求，坚决执行“两准则四条例”，完善财政资金支出、工程项目招投标、国有资产管理等制度，紧盯征地拆迁、扶贫攻坚、惠民利民等重点领域，有腐必惩，有贪必肃，廉洁政府建设有力有效。主动接受人大法律监督和工作监督，自觉接受政协民主监督、职能机关专门监督、社会公众监督和新闻舆论监督，办理人大代表建议74件、政协提案139件。外事侨务、机构编制、审计统计、粮食供销、物价质监、老龄妇幼、人防气象、史志档案等工作也取得了新成效。

在经济发展环境依旧复杂多变的情况下，取得这些成绩实属不易。这是市委坚强领导、市人大市政协全力支持的结果，是全市各族人民攻坚克难、苦干实干的结果。在此，我代表市人民政府，向关心、支持张掖发展的社会各界人士，向奋战在各条战线的人大代表、政协委员、干部群众，向各民主党派、工商联、人民团体、中央和省属驻张单位，向离退休老同志、驻张部队、武警官兵、公安干警表示衷心的感谢并致以崇高的敬意！

我们也更加清醒地看到，经济社会发展和政府工作中还存在不少困难和问题。主要表现在：经济发展中积累的结构性矛盾和制约性因素有叠加之势，经济下行压力依然很大；民间投资意愿不强、动力不足、活力不够的问题比较突出，项目建设和产业培育面临巨大的投融资难题；实体企业杠杆率高、银行不良率高、非法金融活动发生率高相互交织，防范金融风险、优化金融环境和扩大有效投资的任务十分艰巨；企业经营比较困难，产业性投资明显不足，财源培植乏力，财政增收空间小和刚性支出大的矛盾非常突出；城市学校班额大、幼儿入园难、基层医疗单位人才匮乏、供热供水品质不高等方面的民生短板亟待弥补；政府一些部门和干部不愿担当、不敢担当、不会担当的问题比较突出，庸政懒政怠政的问题依然存在，政府效能建设还需持续加强。这些问题，我们一定高度重视并将采取针对性措施逐步加以解决。

关于2019年政府工作

2019年，是决胜全面建成小康社会的关键之年。政府工作的总体要求是：坚持以习近平新时代中国特色社会主义思想为指导，深入贯彻落实习近平总书记视察甘肃重要讲话和“八个着力”重要指示精神及中央、省委省政府和市委一系列决策部署，坚持稳中求进工作总基调，坚持新发展理念，坚持深化市场化改革、扩大高水平开放，统筹生态环境保护与经济高质量发展、脱贫攻坚与乡村振兴、重大风险防范与民生保障改善，全力稳就业、稳金融、稳外贸、稳外资、稳投资、稳预期，推动全市经济社会各项事业迈上新台阶。

经济社会发展主要预期目标是：生产总值增长6%左右。固定资产投资增长10%以上。社会消费品零售总额增长7.5%。一般公共预算收入增长5%。城镇、农村居民人均可支配收入均增长8%左右。单位生产总值能耗、主要污染物排放等约束性指标控制在省上下达指标内。

重点抓好以下七个方面的工作：

（一）构建生态产业体系，高质量发展迈出新步伐

牢牢把握高质量发展要求，以绿色发展崛起为目标，以十大生态产业为支撑，以机制创新和科技创新为动力，以重大项目为载体，推动传统产业转型升级，加快新兴产业引进培育，着力构建符合绿色发展要求、切合张掖市情实际、富有生机活力的生态产业体系。

以打造国际化旅游目的地为目标，着力提升旅游文化产业质量效益。把旅游文化产业作为绿色发展崛起的主攻方向，坚持推进全域旅游、旅游文化体育医养融合发展、低门票价格倒逼企业开发选择性服务“三大战略”不动摇，把延链补链产业项目建设和管理服务水平提升作为重中之重，坚定不移推动旅游文化产业向国际化方向迈进。持续深化“四大行动”，确保丹霞5A级景区通过验收、丹霞世界地质公园通过评审，推进甘州芦水湾生态旅游景区、肃南裕固族度假区创建省级和国家级生态旅游度假区，新创建4A级景区2家以上。紧盯演艺剧目、旅游商品开发及景区游乐项目、户外运动基地、餐饮购物街区等重点领域，持续推进延链补链重点项目建设，开工建设裕固风情园、丹霞轨道交通、民乐文化产业园等22个重点项目，建成丹霞口民俗文化旅游村盛世大剧场等项目，完成投资45亿元以上。以“双十双百”为示范引领，抓好村庄环境、民居建设、基础设施、管理服务等重点领域的提升，努力把乡村旅游培育成新的经济增长点。坚持市场化运作机制，精心举办户外品牌赛事，持续提升“张掖·敦煌写经书法”艺术节等旅游文化节会知名度和影响力。加快发展研学旅游，深度开发地质科普、户外拓展、励志教育等旅游产品。创新营销模式,进一步激活冬春季旅游市场。加快推进旅游标准化试点城市建设，不断提升旅游服务质量。力争全年接待游客3500万人次、旅游综合收入达到240亿元以上、直接从业人员达到8万人以上，旅游对经济增长的支撑能力进一步增强。

以“三元双向”循环为基本模式，加快农业转型升级步伐。把现代农业作为生态产业体系的基础性产业，按照做优种植业、做强养殖业、做大食用菌等特色产业的总体布局，着力促进农业废弃物资源化利用，降低农业面源污染，强化农业生产资源支撑，提升农产品品质和农业产业质量效益。继续抓好“四个百万”工程，大力发展“六个区域特色产业”。启动实施肉牛产业五年规划和三年行动计划，带动养殖业提质增效。加快推进现代畜牧业全产业链试点市、畜禽粪污资源化利用整县推进等项目，新改扩建标准化规模养殖场区100个以上，肉牛、奶牛饲养量分别达到100万头和4.5万头。抓好品种选育、基地建设和质量管控，加强高原夏菜品牌建设。加快发展食用菌产业，力争产量达到9万吨以上。按照全省现代丝路寒旱农业“一带五区”特色农业产业布局，坚持“生态优先、定位高端、企业为主”的思路，新发展戈壁农业2.76万亩，力争民乐海升农业二期、临泽中以合作戈壁农业示范园等重点项目落地实施，基本建成南部食用菌、中部外向型蔬菜、北部优质林果三大戈壁农业产业带。全力抓好三生源30万吨尾菜加工生态农家肥、天泽农牧6万吨饲草、山丹爱福农业食用菌加工等重点农业产业化项目建设，完成投资12亿元以上，新增农产品加工能力4.6万吨。开工建设河西制药二期等重点项目，提高道地药材标准化种植和加工水平，持续壮大中医中药产业。大力培育新型市场主体，新创建农民专业合作社示范社120家、示范性家庭农场75家。巩固提升农产品质量安全水平，新认证“三品一标”农产品40个以上，张掖农业的绿色特点、金色品质进一步提升。

以绿色低碳循环发展为导向，力促工业经济趋稳向好。按照主导产业提质、新兴产业扩容、传统产业增效的思路，坚定不移走好资源循环、企业聚集的生态工业发展路子。持续完善“2+4”工业园区支撑体系，推行“多规合一”，集成配套基础设施，提高投资强度和投入产出比。坚持抓主抓重抓大，全力推进花草滩循环经济产业园、凹凸棒石科技产业园、滨河集团酒文化产业园等“八个生态型产业化项目”，构建生态工业体系。加快智能终端产业科技城建设，引进更多高新企业入园发展，促进先进制造业优化升级。加快推进昆仑生化公司“出城入园”和木材加工企业搬迁改造集约生产，提升产业聚集效应。加快建设丰聚能源天然气储配管网设施及供应、甘州谷电储能等清洁能源产业项目，全力推进兰标生物生态厕所暨功能性肥料、前沿生物复合材料等节能环保产业项目建设，力争开工建设首途新能源3万吨废煤焦油综合利用、危废固废处置及资源化利用等清洁生产项目，提高资源利用效率。积极推动军民融合产业发展，加快建设通用航空产业园、航天九院无人机组装测试等重点项目。依托智慧城市建设，积极培育数据信息产业。大力实施“十强双百”企业振兴计划，全面落实扶持政策，助力企业做大做强做优。完善领导干部帮扶企业和联系企业家制度，帮助企业解决困难问题，推动停产企业复产、减产企业达产、小微企业升规入库,新增规上企业10户以上。

以通道物流产业为重点，提升现代服务业发展水平。着眼健全体系、完善功能、规范秩序、提升效能，实施城区物流体系发展规划，巩固拓展通道物流产业。按照“堵违规、建正规，以堵促建、以建带疏，建管并举、做大做强”的思路，打好物流市场秩序整治攻坚战，着力解决物流仓储大量违建运营和安全隐患突出问题。制定出台物流产业发展支持政策，基本建成G30高速东出口公路港物流园、张掖经济技术开发区物流园和张掖火车站铁路物流园，完善提升张掖绿洲、传化公路港等物流园服务功能。发展壮大本土冷链物流骨干企业，大力培育第三方冷链物流，创新应用新技术新业态新模式，全面提升冷链物流水平。加快农产品市场体系建设，新建嘉宏果蔬产品产地批发市场和4个农贸市场。推动电子商务与一二三产业深度融合，积极建设大宗农产品电商交易平台。改造提升餐饮住宿业、文化娱乐业等传统服务业，加快发展以金融服务、健康养老、商务会展、信息咨询等为重点的新兴业态，补齐服务业发展短板。

（二）实施乡村振兴战略，统筹城乡协调发展开创新局面

抢抓乡村振兴战略机遇，突出城乡统筹发展，坚持以城带乡、以乡促城、城乡联动、整体推进，促进城乡经济社会一体化发展。

加快乡村振兴示范建设。以乡村振兴战略统揽农业农村工作，以“两带四区四线”示范建设为重点，建成新墩双塔、板桥红沟、南华南岔等美丽乡村示范村50个以上，推动乡村振兴逐步向整体推进、全域提升扩展。统筹推进农村人居环境整治、生态环境建设、全域旅游发展、农村“三变”改革等重点工作，高标准建设一批示范带、示范区。加快恒大温泉小镇、通航小镇、速展特色街区等文化旅游小镇和田园综合体建设，增强乡村旅游在实施乡村振兴战略中的引领作用。纵深推进农村人居环境整治三年行动计划，全面抓好垃圾污水处理、厕所革命、村容村貌提升等工作，力争30个城郊村污水接入城市管网，推动环境整治规范化、长效化。

增强城市聚集辐射功能。强化规划刚性约束，修编实施《张掖市城市总体规划》和历史文化名城保护等专项规划，完善城市空间规划体系。以创建国家园林城市为抓手，加快城市道路、市政、园林绿化等基础设施建设。新建东二环路和滨河新区永固路、文昌路等13条道路，改建临泽北路、滨湖路、玉关路等11条道路，着力打通断头路、畅通人行道，进一步完善路网框架。实施滨河新区及老城区主要道路、重点建筑亮化工程，启动建设胡家园子等5个城区小公园，推进市区及五县城区便民市场、停车场、公厕建设，建成临泽、山丹、民乐城区垃圾压缩中转站，提升城市形象。实施市污水处理厂扩容及提标技改、高台再生水利用等重点项目，提高城市承载能力。加强供热能力建设，持续扩大集中供热覆盖面，强化政府监管，提升供热质量。续建5个出城口提升改造工程，实施张靖公路、张莺公路等3个出城口提升改造工程。坚持“以地价控房价、以低价促消费、以消费带投资”，推进租购并举改革，稳定合理低价，扩大住房消费，培育健康的房地产市场，让老百姓想买房、买得起房。坚持建管并重，加强城市管理，积极探索大数据管理和城管综合执法新模式，增强市民参与城市管理、爱护城市设施、珍视城市荣誉的意识，共创张掖文明开放城市新形象。

提升城乡公共服务水平。坚持教育优先发展战略，实施第三期学前教育三年行动计划，新建改扩建公办幼儿园9所，提高普惠性幼儿园办学水平，着力解决“入公办园难、入民办园贵”问题。积极推进农村小规模学校、寄宿制学校建设及厕所和供暖设施改造等全面“改薄”项目，实施高中薄弱学校改造、职业教育基础设施建设等教育现代化推进工程，完成投资3.9亿元。积极推进培黎职业学院建设，着力构建中高职一体化贯通机制。深入推进医药卫生体制改革，加大重点传染病、慢性病防控工作力度，提高全民健康素养，创建全国幸福家庭活动示范市。确保市第二人民医院一期项目规范运营，二、三期项目年内建成投用。加快甘州、临泽、高台、山丹人民医院及甘州区妇幼保健院业务用房等项目建设进度，持续改善提升基层医疗设施水平。深化国家扩大文化消费试点城市建设，确保通过验收。积极推进张掖广电传媒中心、市非遗保护传承中心、临泽城市文化综合体、山丹“七馆”、民乐“五馆”等项目建设，不断完善基层文化服务功能。加大精品剧目创作演出力度，打造特色文化品牌，持续提升公共文化服务水平。着力培育科技创新主体，搭建科技创新平台，推动科技成果转化。大力实施张掖奥体中心及城乡体育场、足球场、全民健身中心等体育惠民工程，完善全民健身服务体系，以全民健身促进全民健康。深入开展新时代精神文明创建活动，大力践行社会主义核心价值观，提高全民道德文明素养。

（三）加大生态保护力度，全域环境质量实现新改善

牢固树立“绿水青山就是金山银山”理念，把改善生态环境作为最普惠的民生福祉、最坚实的发展基础，持续用力，久久为功，不断满足人民群众日益增长的美好生态环境需要。

巩固提升整治成果。牢牢扛起政治责任、主体责任、监管责任，全面完成祁连山自然保护区内矿业权项目和水电站退出等后续工作，确保祁连山生态环境突出问题整改不留后患、黑河湿地生态环境问题全面整改到位。紧盯中央、省级环保督察及省级行业主管部门督查反馈跨年度整改问题，加大力度、全力推进，确保整改到位、逐项销号。坚持实行县区月检查、市级行业主管部门双月巡查、台帐化管理和常态化督查制度，开展“回头看”“再整治”，巩固提升生态环境问题整改整治成果，提标提质，严防反弹。举一反三，加强对各类自然保护区、环境敏感区、生态功能区的常态化监管和问题的即查即改，确保全域生态环境得到有效保护。围绕生态保护与发展的突出矛盾，探索制定地方标准、准入政策，完善保护制度体系。

全面加强生态建设。加快创建生态文明建设示范市，认真组织实施三北防护林、新一轮退耕还林还草、沙化土地封禁保护等重大生态项目，争取实施重要生态系统修复和保护、北郊湿地恢复与治理、双泉湖湿地公园等生态保护修复项目，全面完成“两转四退四增强”治理提升三年行动计划目标任务，持续改善全域生态环境。加快推进山水林田湖草生态保护修复及祁连山保护修复综合治理规划年度项目建设，完成投资12亿元以上。坚持“造林”与“造景”相结合，“防护林”与“景观林”相融合，重点生态工程与农田林网、村庄绿化相配套，完成以“一园三带”造林绿化示范建设为重点的营造林26万亩，带动完成大规模国土绿化56万亩。积极配合推进祁连山国家公园体制试点工作。建设森林资源综合监管数据化平台，完善森林资源监测网络，形成立体化、动态化监管格局。加强自然资源统一确权登记工作，强化生态文明建设目标评价考核，加大领导干部自然资源资产离任（任中）审计力度，加快构建推进生态文明建设常态化机制。

高效治理环境污染。探索创新应用大数据等技术手段推动科学治污，全力以赴打好“蓝天”“碧水”“净土”保卫战。深化大气污染防治，持续推进农村炕烟炉烟、秸秆垃圾焚烧、燃煤锅炉、道路扬尘等主要污染源专项整治，巩固提升空气质量。持续加强水环境保护，加快实施沿河村镇污水集中收集处理等流域水污染防治综合整治工程，完成县级及以上集中式饮用水水源地规范化建设。严格落实河湖长制，加强水资源管理，继续实施地下水超采区治理，启动山丹河及黑河城区段西河生态治理，推进水环境持续向好。积极开展土壤污染防治，实施土壤污染治理与修复技术应用试点项目，完成民乐铬盐厂铬污染土壤修复年度任务。全面实施农业农村污染治理攻坚战行动计划，推动农村生态环境持续好转。严格执法监管，始终保持打击环境违法行为高压态势。加强宣传，倡导绿色低碳发展方式和生活方式，增强全民环保意识。

（四）加快基础设施建设，补齐发展短板取得新突破

聚焦重点领域和薄弱环节，加大基础设施领域补短板力度，不断完善基础设施和公共服务，着力提升基础设施供给质量，更好发挥有效投资的关键性作用，持续巩固经济回稳向好态势。

紧盯短板弱项谋划争引基础设施项目。聚焦公路交通、农田水利、清洁能源、扶贫攻坚、生态环保、社会民生等基础设施领域突出短板，对接经济发展和民生需要，谋划储备基础设施领域补短板重点项目564项，概算投资1400亿元。坚持实行重大项目“五个一”推进机制和“双告知双联系双评价”制度，紧扣项目申报、资金筹措、用地保障等关键环节，积极协调服务，强化督查调度，努力提高项目开工率、资金到位率、建成投产率，力争全年开工建设400项以上。

加快重点领域基础设施建设进度。加快张扁高速、G213肃南至祁连二级公路建设进度，争取G312张掖至临泽一级公路、S18张肃公路张掖至康乐段PPP项目落地实施，确保S236新天至马蹄寺、S315花草滩至焉支山、G30丹霞互通立交建成投入使用，开工建设山丹新城区客运站，积极推进S301北部大通道、马场四场至扁都口段高等级公路等项目前期工作，配合推进兰州至张掖铁路三四线前期工作。实施好金山水库等戈壁农业重点水源工程和中小河流治理、病险水库除险加固等重点水利工程，力争开工建设高台山水河、民乐小堵麻水库和甘州区酥油口下库。整理土地9.7万亩，新建高标准农田28万亩。加快张掖750千伏、甘州330千伏输变电工程项目建设，统筹推进“低电压”综合治理、三相动力电延伸覆盖等工作。加快全省5G网络试点示范市建设，新建通信光缆3000公里，全市出口带宽达到2400G。

着力拓宽项目建设投融资渠道。统筹采取有效措施保障合理融资需求，推动基础设施领域重点项目顺利实施。全面落实支持民间投资的各项政策措施，鼓励引导民间资本投资建设重大基础设施项目。持续优化金融生态环境，支持金融机构化解不良贷款，加快引行入张步伐，引导金融机构创新金融产品，扩大信贷规模。主动对接社会资本，拓宽项目融资渠道，规范有序推进PPP模式。加快落实省政府出让国有资源资产和资产证券化补充基础设施项目资本金的各项措施，积极争取省政府设立的基础设施、产业基金在张掖落地。下功夫盘活存量资产，通过债券、股权、股债结合、基金等多种形式，努力为在建项目和补短板重大项目提供融资支持。坚持“主攻新三板、争取上主板、扩大股权融资”的多层次资本市场培育策略，提升企业直接融资能力。深化政府投融资平台与金融机构合作，加快市城投、山水文旅集团等国有投融资平台债券发行工作，保障项目建设资金需求。

（五）切实保障改善民生，人民生活水平得到新提高

坚持以人民为中心的发展思想，把提升人民群众的获得感、幸福感、安全感作为一切工作的导向，加强和创新社会治理，提高社会保障能力，使发展成果更多、更好、更公平地惠及全体人民。

坚决打赢脱贫攻坚战。持续做好贫困人口精准识别精准退出工作，加强贫困人口动态管理，严把退出关口，进一步夯实精准帮扶基础、产业脱贫基础，巩固提升脱贫成果，确保剩余贫困人口全部脱贫、脱贫人口稳定增收不返贫。完善并严格落实“一户一策”精准脱贫计划，鼓励贫困户积极参与“三变”改革，加快建设一批以农畜产品深加工、仓储物流为主的“扶贫车间”；积极培育新型农业经营主体，加强农民专业合作社规范化建设，吸纳贫困群众就地就近稳定就业，提升脱贫致富内生动力。坚持脱贫攻坚与乡村振兴统筹发力，推进贫困村基础设施和公共服务设施建设。加快推进农村金融综合服务室建设，强化脱贫金融保障。强化扶贫资金使用管理，落实监管责任,提高使用效益。持续开展扶贫领域作风问题专项整治，以严明的纪律、务实的作风保障扶贫攻坚各项任务有效落实。

提升社会保障水平。坚持把就业作为最大民生，落实各项创业就业扶持政策，持续推进就业创业“三大计划”，城镇新增就业2.4万人以上，城镇登记失业率控制在4%以内，高校毕业生就业率保持在85%以上，输转劳动力28万人以上。健全落实保障农民工工资考核问责长效机制，切实维护农民工合法权益。按照兜底线、织密网、建机制的要求，深入实施全民参保计划，精准扩大社会保障覆盖面，加快构建可持续、多层次的社会保障体系。统筹完善城乡社会救助体系，推进社会救助制度与扶贫开发政策有效衔接，做到应保尽保、应扶尽扶。改造棚户区15353套（户），发放住房租赁补贴4160户。健全农村留守儿童关爱服务体系和保护救助体系。全面放开养老服务市场，加快公办养老机构改革，鼓励社会力量兴办养老机构，建成市老年人康复中心，支持甘州区全国综合养老示范基地扩大规模、规范运营。建成投用市社会福利院、市救助管理站暨未成年人社会保护中心，立项建设民乐、高台残疾人托养中心，使弱势群体得到更多保护和关爱。

创新和加强社会治理。全面落实“七五”普法规划，增强全民法治意识，推进依法治市进程。坚持自治、法治、德治相结合，创新基层治理和服务方式,着力优化基层社会治理体系。统筹推进城乡社区建设，完善服务设施和功能配置，提升治理能力。加强机构和职能融合，构建应急管理、安全生产、防灾减灾于一体的应急管理体系，提升灾害事故防治和应对处置能力。严格落实“党政同责、一岗双责、失职追责”安全生产责任制，有效防范和遏制重特大事故发生。深入宣传落实张掖首部地方实体法《张掖市城市道路交通管理条例》，持续提升城市交通管理水平。实“四个最严”“四有两责”要求，全面提高食品药品安全监管水平。深入创建“全国双拥模范城”，扎实做好退役军人服务管理。全面贯彻党的民族宗教政策，促进民族团结、宗教和顺。健全完善多元矛盾纠纷排查调处体系，构建“大信访”格局。持续推进平安张掖建设，深化扫黑除恶专项斗争，严厉打击毒品违法犯罪、涉众型经济犯罪和网络电信诈骗犯罪，维护社会和谐稳定。

（六）持续优化营商环境，区域经济发展形成新活力

坚持把改革创新贯穿于经济社会发展各个领域，着力破除体制机制障碍，激发市场主体活力，降低创业创新风险与成本，增添经济发展动力。

深化重点领域改革。深入总结试点经验，全面推进农村“三变”改革，大力培育龙头企业、农民合作社等承接主体，搞好“三资”确权量化，完善细化折价入股办法，建立科学合理的利益联结机制、收益分配机制和风险防控机制，因地制宜培育发展特色产业，切实让村集体和农户受益。完善农村“三权分置”制度，更多释放农村发展活力。全面实施预算绩效管理改革，继续压减“三公”经费等一般性支出，强化预算约束，倾力民生保障，持续提高财政支出绩效。加强政府债务管理，规范各级政府举债融资行为，坚决遏制隐性债务增量。启动实施县区机关事业单位农（林）场改革，明晰产权关系，理顺经营机制，促进资产保值增值和有效利用。深化国企国资改革，进一步完善市属国有投融资公司法人治理结构。持续推进创业创新平台建设，切实提升服务功能和运营水平，进一步巩固提升“双创”成果。

支持民营经济发展。始终坚持“两个毫不动摇”“三个没有变”，坚定不移推动民营经济高质量发展。细化量化政策措施，完善政策执行方式，推动减税降费、放宽准入等各项政策落实落地，提振民营经济发展信心。强化银政企对接合作，继续加大金融支持力度，创新金融服务，管好用好企业应急周转金和融资担保基金，努力缓解民营企业融资难、融资贵及形成不良信用记录等问题。对优惠政策不落实、招商承诺不兑现、“新官不理旧事”等重点问题进行挂牌督办，切实解决一批影响民营企业投资和民营经济发展的突出问题。创新规范执法方式，避免简单化，不搞“一刀切”，严防因执法不当影响民营企业正常生产经营。关心民营企业发展、民营企业家成长，健全完善激励机制，切实保障企业合法经营和企业家合法权益，激发民营企业和企业家的创新创造活力。

加大招商开放力度。坚持“引进来”和“走出去”并重，积极构建开放型经济发展新格局。深入实施“引强入张”和精准招商行动，大力开展招商引资、招展引会、招才引智，持续引进投资规模大、产业配套强、附加值高的项目。以循环农业、旅游文化产业、智能终端制造、通用航空、凹凸棒新材料开发等为重点，主要领导挂帅，企业联动发力，主动上门对接，增强招商实效。强化项目落地服务，营造重商亲商安商的良好环境，力促招商引资到位资金稳定增长。深度融入“一带一路”建设，务实推进向西开放、向东融入。积极培育壮大外贸市场主体，培养引进外贸人才，支持有条件的企业在霍尔果斯、广西凭祥口岸兴建仓储设施及产品展厅，设立境外销售网店，争取开展中欧班列集拼集运业务，扩大优势产品出口规模。积极推进对外劳务合作，探索与中西亚国家开展农业合作。突出绿色生态主题，全面提升“张交会”国际化、专业化、市场化水平，打造一流品牌展会。

（七）加强政府自身建设，忠诚担当干事创业展现新作为

始终保持敢于担当、勇于作为的精神状态，忠实履行人民政府为人民的职责，全面提升行政效率和施政水平。

着力提升政府行政效能。认真贯彻落实中央及省委省政府和市委决策部署，以市县级党政机构改革为契机，进一步优化职能配置，构建系统完备、科学规范、运行高效的政府机构职能体系。持续深化“放管服”改革，“减”字当头，简化审批程序，再造工作流程，压缩办理时限；“优”字为先，提升人员素质，改进服务态度，为企业和群众提供优质服务。全面落实国家法治政府建设实施纲要，坚持法定职责必须为、法无授权不可为，依法全面履行政府职能。全面推行行政执法公示制度、执法全过程记录制度、重大执法决定法制审核制度，切实保障人民群众合法权益，维护政府公信力。严格落实政府重大决策法定程序，提高行政决策科学化、民主化、法治化水平。依法接受人大及其常委会工作监督和法律监督，主动接受政协民主监督，认真听取各民主党派、工商联、无党派人士和人民团体意见，提高人大代表建议和政协提案办理质量，让政府工作更好体现民意。

坚持不懈加强作风建设。把落实执行作为作风建设的核心和关键，增强干部“要干”的责任，激发干部“想干”的热情，创造干部“能干”的氛围，实干为本、苦干为乐、干成为荣，以各级干部的担当作为确保任务目标的全面完成。坚决贯彻落实中央八项规定及实施细则精神，严格执行省委“七破七立”要求和省政府“十不准”规定，统筹规范督查检查考核工作，严防“四风”问题反弹回潮，推动行风政风作风持续好转。完善“市级政府决策担当，县区部门落实执行”工作机制，坚决杜绝政府决策环节庸政懒政问题发生，为重点任务的落实和重大项目的推进争取时机、创造条件。健全市政府议事会议制度，每季度研究一个事关全局的重大问题，协调解决突出矛盾，明责赋权加快落实。完善绩效考核评价体系，用好正向激励机制，落实容错纠错制度，加大督查问责力度，坚决整治不作为、慢作为、乱作为等行为。大力弘扬立说立行、马上就办的作风，促进各项决策部署落地生根。

持之以恒推进廉洁政府建设。严格落实全面从严治党主体责任，持续加强政府系统党风廉政建设。强化廉洁自律意识，筑牢拒腐防变堤坝，守住从政做事底线。紧盯土地矿权出让、项目招投标、工程建设等重点领域，完善优化制度体系，深化专业化检查督查，提高公开性、透明度，防范政府权力滥用和少数人以权谋私。健全风险预警和权力运行监控机制，加强审计监督，推行政府投资项目造价审计全覆盖，严控成本，严防权力寻租。严查违纪违法，营造干部清正、政府清廉、政治清明的良好环境。

各位代表！张掖是我们共同的故土家园，是130万张掖人民安身立命、生生不息的热土宝地，建设丝路明珠金张掖、开创幸福美好新生活，既是我们的愿景和向往，也是我们的责任和使命。让我们在习近平新时代中国特色社会主义思想指引下，在省委省政府和市委的坚强领导下，聚全市之力，集全民之智，开拓创新，砥砺前行，奋发有为，决胜全面建成小康社会，以优异成绩迎接中华人民共和国成立70周年！
